# Auto-FPFA: An Automated Microscope for Characterizing Genetically Encoded Biosensors

**DOI:** 10.1038/s41598-018-25689-x

**Published:** 2018-05-09

**Authors:** Tuan A. Nguyen, Henry L. Puhl, An K. Pham, Steven S. Vogel

**Affiliations:** Laboratory of Molecular Physiology, National Institute on Alcohol Abuse and Alcoholism, National Institutes of Health, 5625 Fishers Lane, Rockville, Maryland USA

## Abstract

Genetically encoded biosensors function by linking structural change in a protein construct, typically tagged with one or more fluorescent proteins, to changes in a biological parameter of interest (such as calcium concentration, pH, phosphorylation-state, etc.). Typically, the structural change triggered by alterations in the bio-parameter is monitored as a change in either fluorescent intensity, or lifetime. Potentially, other photo-physical properties of fluorophores, such as fluorescence anisotropy, molecular brightness, concentration, and lateral and/or rotational diffusion could also be used. Furthermore, while it is likely that multiple photo-physical attributes of a biosensor might be altered as a function of the bio-parameter, standard measurements monitor only a single photo-physical trait. This limits how biosensors are designed, as well as the accuracy and interpretation of biosensor measurements. Here we describe the design and construction of an automated multimodal-microscope. This system can autonomously analyze 96 samples in a micro-titer dish and for each sample simultaneously measure intensity (photon count), fluorescence lifetime, time-resolved anisotropy, molecular brightness, lateral diffusion time, and concentration. We characterize the accuracy and precision of this instrument, and then demonstrate its utility by characterizing three types of genetically encoded calcium sensors as well as a negative control.

## Introduction

Fluorescence microscopy has been extensively used as a tool for monitoring biological molecules of interest that can be tagged with a fluorophore^1–3^. With proper instrumentation, several aspects of fluorescence can be monitored^4^, including emission intensity, absorption spectrum, emission spectrum, lifetime, anisotropy, concentration, molecular brightness, and the lateral & rotational diffusion of the fluorophore. The discovery of genetically encoded Green Fluorescent Protein (GFP)^5^ and the rapid development and bioengineering of genetic variants of GFP^6–9^ and related proteins (FPs) has led to the development of genetically encoded biosensors that typically have one or more FPs attached to a protein moiety designed to change its structural conformation in response to a biological parameter of interest, such as free calcium^10^. By diligent and creative genetic engineering, bio-parameter induced structural changes will alter the photo-physics of attached fluorophores, and these changes in the photo-physics can be monitored with appropriate instrumentation to estimate changes in the bio-parameter of interest. Unfortunately, the majority of biosensors currently available work by detecting only changes in the intensity of one or more fluorophores, while information imbedded in the other modes of fluorescence have remained unexplored and untapped. We believe that this limitation on the design pallet available to biosensor developers arises primarily because of a lack of instrumentation that can 1: rapidly & reliably, and 2: simultaneously measure multiple photo-physical changes in biosensors. Automated microscopes^11–15^ have been developed to address the first issue, but robotic systems that can concurrently monitor multiple photo-physical properties have not been reported. In this paper, we describe the design, construction, and utility of an automated multi-modal microscope that simultaneously measures fluorescence intensity (photon count), lifetime, time-resolved anisotropy, molecular brightness, concentration, and lateral diffusion.

## Results

### Design and implementation of Auto-Fluorescence Polarization and Fluctuation Analysis (auto-FPFA)

We previously designed a microscope that used two-photon linearly polarized pulsed excitation in conjunction with time-correlated single photon counting (TCSPC) instrumentation to measure fluctuations in the intensity of parallel, I_||_(t), and perpendicular, I_⊥_ (t), polarized fluorescent emissions over time, as well as the fluorescence intensity decay of the parallel, I_||_(∆t), and perpendicular I_⊥_ (∆t), polarization, as a function of time after an excitation laser pulse^16–19^. I_||_(t), and I_⊥_ (t) were cross correlated for fluorescence correlation spectroscopy (FCS) analysis to reveal bio-photonic attributes of a sample such as count-rate, molecular brightness, concentration, and *τ*_D_^20–22^. I_||_(∆t), and I_⊥_ (∆t) are used to calculate the fluorescence lifetime decay of the sample as well as its time-resolved anisotropy decay^11,23–26^. We called this multimodal approach *Fluorescence Polarization and Fluctuation Analysis* (FPFA)^16^. In this paper, we set out to design an automated variant of FPFA to autonomously measure up to 96 samples. The major design challenges for implementing FPFA microscopy is that FCS microscopy signal to noise is optimized by using high numerical aperture (NA) objectives that minimize the excitation volume^27–32^. In contrast, time resolved anisotropy measurements are best implemented using low NA objectives to minimize depolarization^33,34^. Furthermore, to build an automated variant of FPFA for micro-titer plates it is advantageous to avoid objectives that require oil or water deposition between the objective and the bottom of the plate as liquids can evaporate, drip or enclose bubbles that can corrupt automated measurements. Finally, for accurate FCS measurements the excitation volume of an objective, as defined by its point spread function^35^, should reside completely within the sample volume. Thus, objectives with long working distance are advantageous, in conjunction with an automated focus mechanism to keep the excitation volume well within the sample volume as an automated X-Y stage moves systematically through all samples.

**Figure 1:**
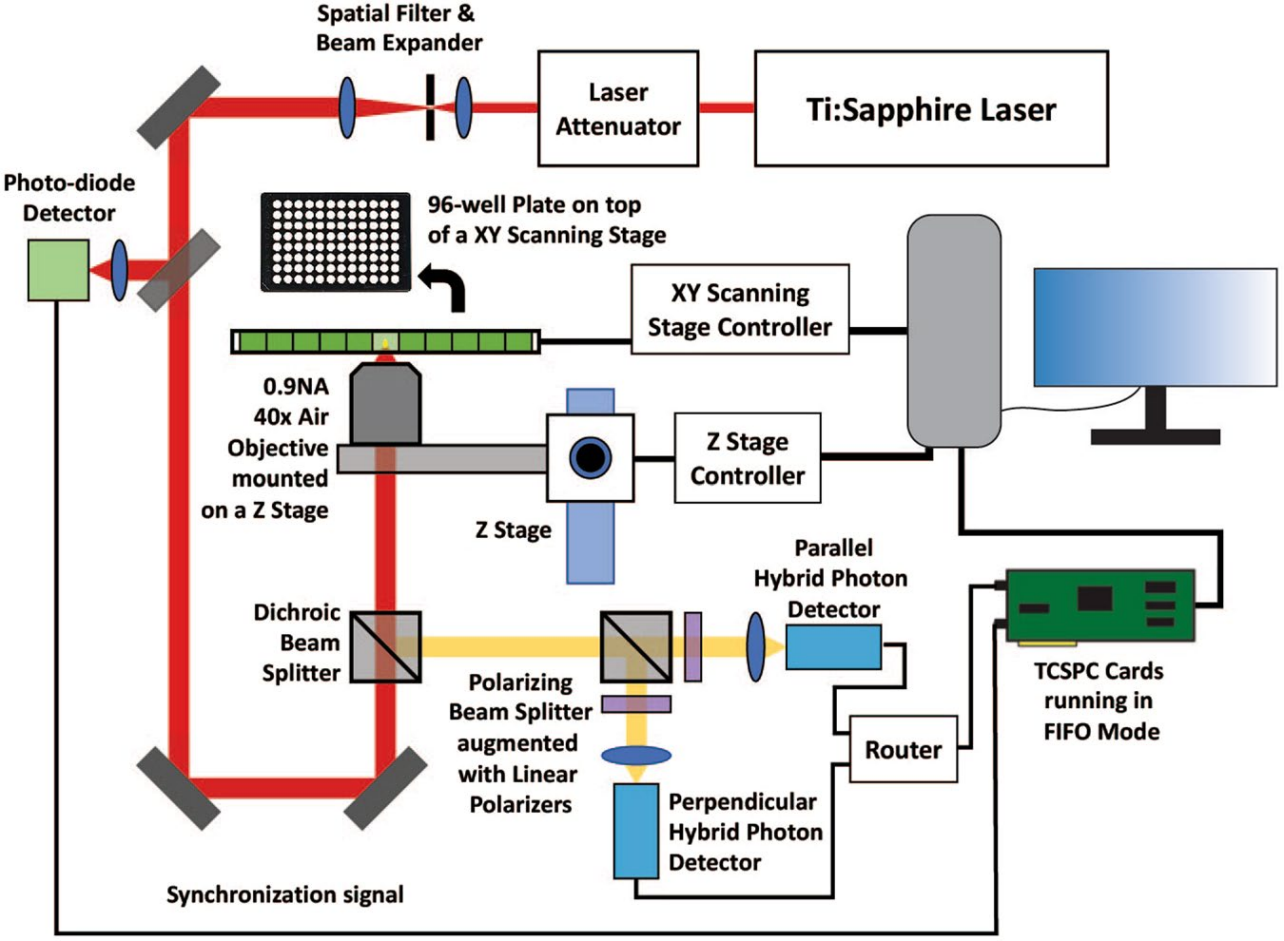
Auto-FPFA schematic. The light path and components used for implementing auto-FPFA. Key components that have been added or modified from previous implementations of FPFA include the integration of a XY-dual axis scanning stage controller, a Z-axis stage controller, and the use of a 0.9 NA 40x air objective.

The design of our implementation of auto-FPFA is illustrated in Fig. 1. An 80 MHz, 70-fs Ti:Sapphire laser (MaiTai eHP, Spectra-Physics) tuned to 950 nm is used for two-photon excitation. The laser is filtered and expanded (KT310, Thorlabs), and then passed through a laser attenuator consisting of near-IR half-wave plate and linear polarizer (100,000:1 extinction ratio, Thorlabs) where the excitation power and polarization at the sample can be adjusted. Next, the excitation beam is directed through a multiphoton long-pass dichroic beamsplitter (FF665-Di02-25 × 36, Semrock) to an air microscope objective (Zeiss 40x 0.9 NA, with back aperture slightly overfilled) that focuses the beam to a diffraction-limited spot (~0.5 μm in diameter) to form the excitation/observation volume (1.7 ± 0.7 fl). Sample fluorescence emanating from the observation volume was reflected by the same dichroic beam-splitter and then filtered by a high throughput two-photon band-pass emission filter (FF01-520/70-25, Semrock). The fluorescence emission is next guided to a polarizing beam splitter augmented with two orthogonally oriented linear polarizers (Thorlabs) to increase the polarization extinction ratio. At the polarizing beam splitter, parallel and perpendicular emitted photons are separated and each signal is detected by its own dedicated HPM-100-40 hybrid detector (Becker & Hickl). The dark count rate for these detectors is 300–600 cps at room temperature. Detected photons were passed to a SPC-130 EM TCSPC card (Becker & Hickl) via a router (HRT-41, Becker and Hickl). For synchronization with excitation pulses a small fraction of the excitation beam was focused onto a fast photodiode (DET10N, Thorlabs). The SPC-130 card records both micro-time (the time between excitation and photon detection) and macro-time (the time between experiment initiation and photon detection) for each parallel and perpendicular photon detected.

Samples are pipetted into a glass-bottom 96-well microplate (Greiner Bio-One). The plate is then attached onto a XY motorized stage (HLD117, Prior Scientific) so that samples can be scanned over the microscope objective. To compensate for imperfections in the flatness of these microplates, a system was created to automatically focus along the Z-axis at each well location. This was implemented by inversely mounting the objective on a post-mountable focus block (MGZ30, Thorlabs) whose fine adjustment knob was attached to a motorized microscope focus controller (MFC1, Thorlabs) to provide computerized Z axis adjustments.

**Figure 2:**
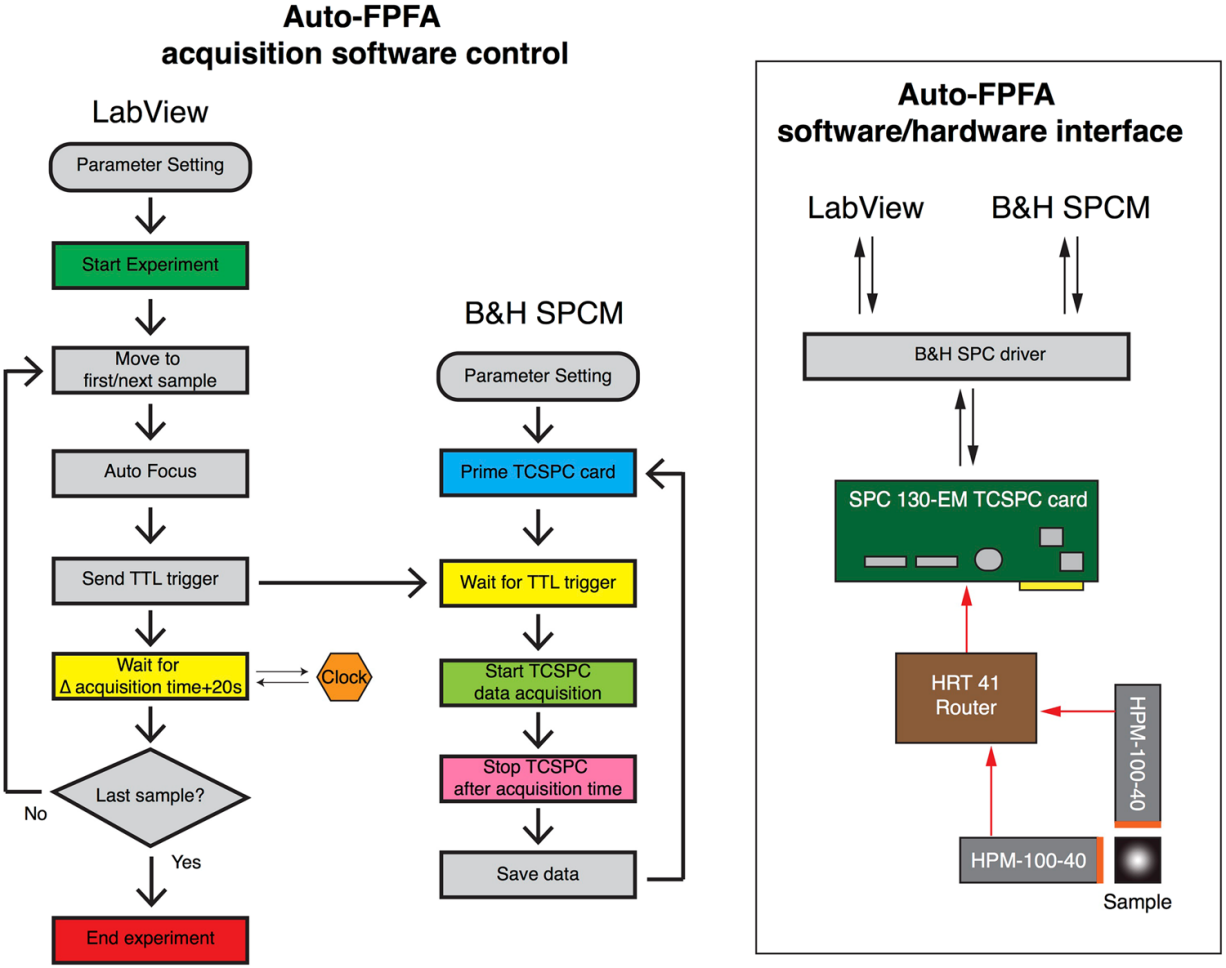
Auto-FPFA acquisition software control and software/hardware interface schematic. Left Panel: Diagram illustrates the Lab View control algorithm, and its integration with B&H SPCM software algorithm. LabView was used as the ‘master’ control data acquisition software, while B&H SPCM software was ‘slaved’ to it. LabView specifically controlled the XY-dual axis scanning stage controller that sequentially steps through the 96 samples in a micro-titer dish, as well as the Z-axis stage controller used for the auto-focus function. The ‘slaved’ B&H SPCM software was used to control the TCSPC SPC-130-EM card for data acquisition and data storage. Right Panel: Diagram illustrates the auto-FPFA software/hardware interface. The central component of this system is the B&H SPC 130-EM TCSPC card. Both LabView and B&H SPCM software communicate (send commands and receive data) with the TCSPC card via a B&H SPC driver. Once activated, the TCSPC card receives data from the two HPM-100-40 hybrid photon detectors via a HRT 41 multiplexing router. The TCSPC card also receives laser pulse timing data from a photo-diode detector (not shown, but see Fig. 1).

SPCM software (Becker & Hickl, Ver. 9.6) running in FIFO mode was used for data acquisition, storage and calculation of time-resolved fluorescence and auto-/cross-correlation functions from micro- and macro-time data, respectively. In parallel, X-Y and Z motorized stages were controlled via a custom LabView (National Instruments, Ver. 2015) program based on the manufacture’s libraries. The coordination between LabView and SCPM software for Auto-FPFA, as well as the software-hardware interface is displayed in Fig. 2.

Prior to initializing an experiment, it is important to perform system calibration, laser power adjustment, background and sample trial measurements (so that the sample’s concentration can be optimized) and the software parameters for both LabView and SPCM software entered into each program. Next, the TCSPC card is primed and put into TTL-triggered waiting mode. To initiate an auto-FPFA experiment the LabView software is started. This moves the microplate so that the first (next) sample is positioned above the objective. Next, the auto-focus sub-routine is launched. The Z-axis controller systematically moves the objective up toward the sample. At each Z-axis location, the fluorescent intensity is recorded and compared to previous measurements to find the Z-axis position that has the maximum fluorescence intensity. Once the optimal Z-axis position is found, a TTL signal is sent by LabView to trigger data acquisition on the TCSPC card. The LabView software enters a waiting period set to be equal to the photon collection time parameter of the TCSPC card, plus an additional 20 seconds so that the SPCM card has sufficient time to acquire and save the data and prime TCSPC card for the next sample well acquisition. This cycle is repeated until all sample wells are scanned.

Two-photon excitation power (at the sample) was typically 9.6 mW to avoid bleaching during acquisition (~150–200 s per well)., Motorized stage components were anchored via Ø1.5” damped posts (Thorlabs) to minimized mechanical vibrations. For each sample, averaged measurements were acquired from three to five repeats. All experiments were performed at room temperature.

### The accuracy & precision of auto-FPFA

**Figure 3:**
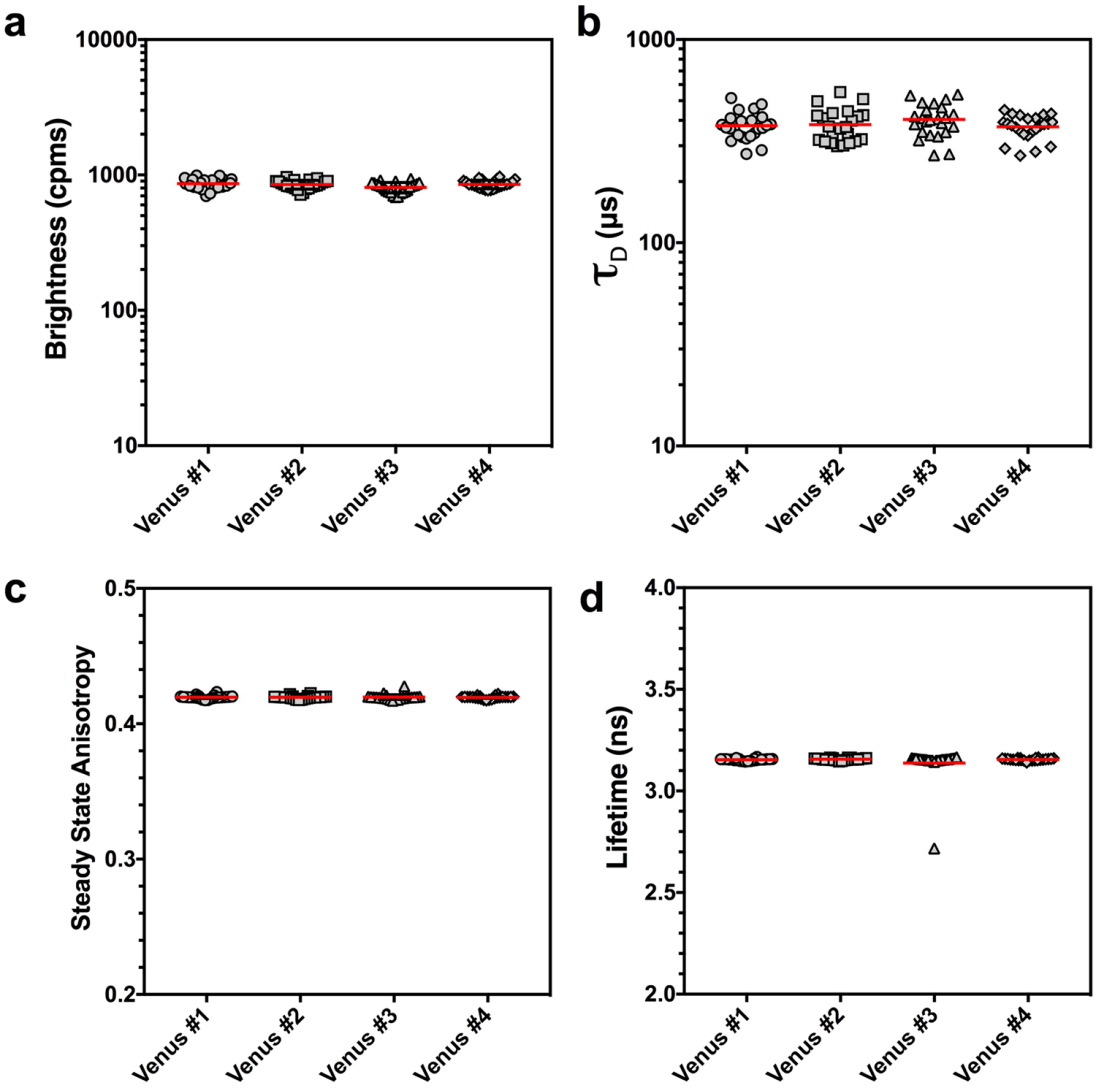
Auto-FPFA accuracy and reproducibility. Auto-FPFA measurements of the molecular brightness (**a**), diffusion time (*τ*_D_, **b**), steady state anisotropy (**c**), and lifetime (**d**) of Venus monomers. Four cell homogenates (circle, square, triangle and diamond symbols) were prepared from cells expressing the Venus fluorescent protein. Replicates of these homogenates were deposited in 24 different wells of a micro-titer dish and all 96-wells were measured overnight by auto-FPFA. Red bars indicate the mean of the 24 replicates of each sample. Note the outlier that is easily detected in Venus sample #3 in panel d.

To characterize the accuracy and precision of auto-FPFA we prepared four homogenates from cells transfected with a DNA construct to express the fluorescent protein mVenus^36^. For each, 24 200 µl replicates were pipetted into wells of a microtiter-dish (making a total of 96 wells) and automated data acquisition was initiated. For each well three 200 s data acquisition periods were acquired and results were averaged. Thus, each well took ~10 minutes for data acquisition and the whole plate required ~16 hours. Because each well of the microtiter dish had mVenus, we used this experiment to characterize the repeatability of our measurements. The molecular brightness for the mVenus replicates are depicted in Fig. 3a and had values of: 863 ± 74, 849 ± 64, 809 ± 72, and 852 ± 61 cpms (mean ± SD, n = 24), and the ensemble mean was 843 ± 70 cpms (mean ± SD, n = 96). A brightness of 843 cpms should have a Poisson counting error of ~29 counts. Thus, the additional error we measure (41 counts) represents 5% of our total mVenus brightness. Diffusion time (*τ*_D_) measurements are depicted in Fig. 3b and had values of: 377 ± 58, 382 ± 71, 405 ± 73, and 372 ± 50 µs (mean ± SD, n = 24) and the ensemble *τ*_D_ value was 383 ± 61 µs (mean ± SD, n = 96). Our error represents ~16% of our total *τ*_D_. The steady-state anisotropy for the mVenus replicates are depicted in Fig. 3c and had values of: 0.419 ± 0.001, 0.420 ± 0.001, 0.420 ± 0.002, and 0.420 ± 0.001(mean ± SD, n = 24) and the ensemble anisotropy value was 0.420 ± 0.002 (mean ± SD, n = 96). Our error represents approximately 0.5% of our signal. The mVenus fluorescence lifetime for the four replicate groups are depicted in Fig. 3d and had values of: 3.154 ± 0.005, 3.156 ± 0.005, 3.137 ± 0.090, and 3.155 ± 0.005 ns (mean ± SD, n = 24) and the ensemble lifetime value was 3.150 ± 0.045 ns (mean ± SD, n = 96). Note that one well from group #3 appeared to be an outlier (panel 3d), and was confirmed by a failure in a D’Agostino & Pearson normality test for group #3 (P < 0.0001, n = 24). Regardless, this sample was included in the analysis above to illustrate that because of the high n-values possible with auto-FPFA, in this case 24 replicates per group, the identification of outliers is simplified, and their impact on group statistics are minimized. Exclusion of this data point resulted in a group #3 lifetime of 3.155 ± 0.005 (mean ± SD, n = 23) from a data set that now passed the D’Agostino & Pearson normality test (P = 0.5763, n = 23), and now an ensemble fluorescence lifetime value for mVenus was 3.154 ± 0.005 ns (mean ± SD, n = 95). The error in our lifetime measurement represents approximately 0.2% of our signal.

**Figure 4:**
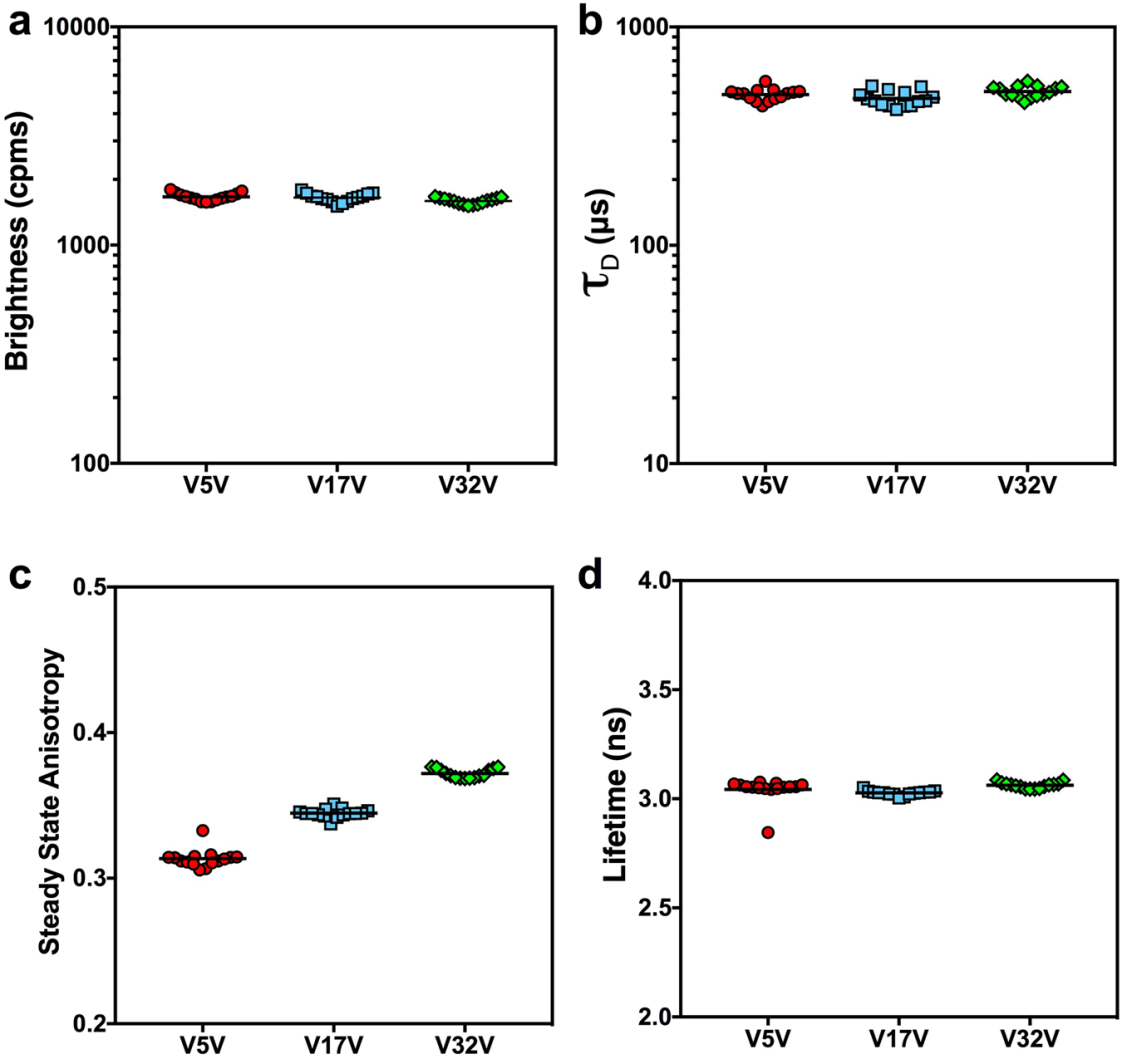
Auto-FPFA can detect subtle structural changes. Auto-FPFA was used to characterize V5V (red circles), V17V (blue squares), and V32V (green triangles). Each Venus dimers was composed of two Venus fluorophores separated by 5, 17, or 32 amino-acid linkers respectively. Auto-FPFA measurements of molecular brightness (**a**), diffusion time (*τ*_D_, **b**), steady state anisotropy (**c**), and lifetime (**d**) of the three types of Venus dimers. For each Venus dimer sample three cell homogenates were prepared and five replicates were measured for each homogenate. Red bars indicate the mean, N = 15 (5 replicates of 3 homogenates for each dimer construct). Note the outlier that is easily detected in the V5V sample in panels c and d. We checked if the location on the micro-titer dish for this outlier was the same as the outlier previously observed in Fig. 3. It was not, indicating that it is unlikely that the outliers resulted from a systematic error in X/Y-axis positioning.

Next, we measured and compared 15 replicates of three different mVenus dimers, V5V, V17V, and V32V, where two mVenus fluorophores are connected by 5, 17, or 32 amino-acid linkers respectively. Venus dimers should have approximately twice the molecular brightness of mVenus monomers, their *τ*_D_ should be larger than mVenus monomers, their fluorescent lifetimes should be similar to the lifetime of the mVenus monomer, but their anisotropy should be lower than the mVenus monomer because of homo-FRET between the fluorophores. Furthermore, because of the distance dependence of FRET we expect V5V to be the most depolarized, V32V to be the least, with V17V having an intermediate anisotropy value. In Fig. 4 we see the auto-FPFA analysis of these three dimers, where panel a shows brightness, panel b *τ*_D_, panel c steady-state anisotropy, and panel d the lifetime. V5V molecular brightness was 1665 ± 68 cpms, V17V was 1650 ± 75 cpms, and V32V was 1593 ± 51 cpms (mean ± SD, n = 15). Thus, V5V was 2.0 ± 0.2 times the brightness of a mVenus monomer, V17V was 2.0 ± 0.2 times the brightness of a mVenus monomer, and V32V was 1.9 ± 0.2 times the brightness of a mVenus monomer. All ratios were consistent with these molecules being mVenus dimers. The *τ*_D_ for V5V was 490 ± 31 µs, V17V was 471 ± 37 µs, and V32V was 507 ± 30 µs (mean ± SD, n = 15). All were larger than the *τ*_D_ of mVenus monomers, 383 ± 61 µs, as expected. ANOVA of *τ*_D_ values revealed that V17V was statistically different from V32V (p = 0.0019). Presumably the hydrodynamic volume of these constructs, while similar, are not identical, and suggests that linker length can have an unpredictable impact on *τ*_D_. The lifetime of V5V was 3.05 ± 0.06 ns, V17V was 3.03 ± 0.01 ns, and V32V was 3.06 ± 0.01 ns. All decayed slightly faster than the lifetime of mVenus monomers, 3.154 ± 0.005 ns, but the difference was substantially less than the instrument response function of the hybrid detectors used (~140 ps). In contrast, the steady-state anisotropy of V5V was 0.313 ± 0.006, V17V was 0.345 ± 0.003, and V32V was 0.372 ± 0.003 (mean ± SD, n = 15). All were lower than the monomer anisotropy (0.420 ± 0.002), and as expected, V5V was the most depolarized, and V32V the least.

**Figure 5:**
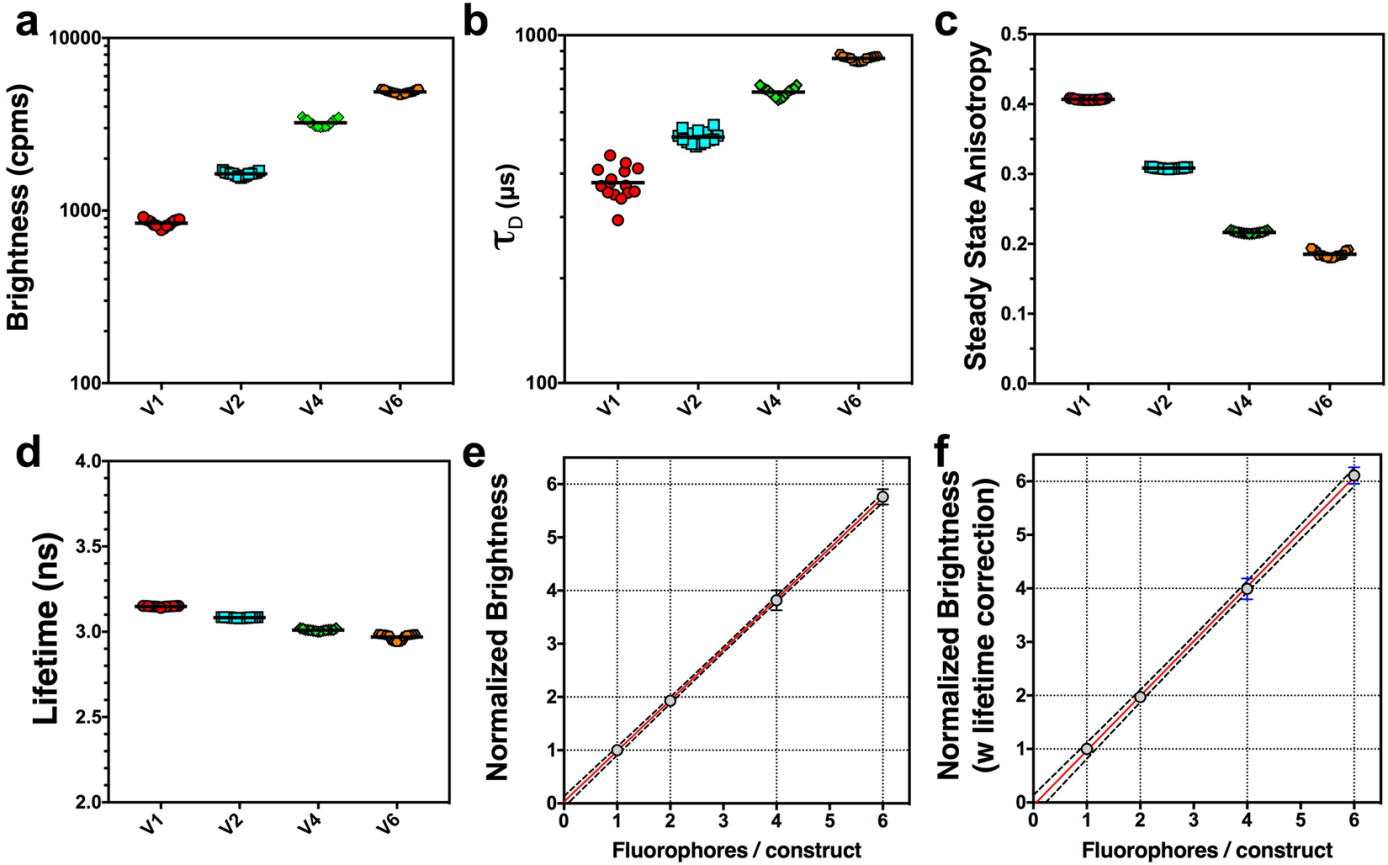
Auto-FPFA can detect structural changes caused by concatemerization. Auto-FPFA was used to characterize and compare Venus monomers (V1, red circles), Venus dimers (V2/V5V, blue squares), Venus tetramers (V4, green diamonds), and Venus hexamers (V6, orange hexagon). Auto-FPFA measurements of molecular brightness (**a**), diffusion time (*τ*_D_, **b**), steady state anisotropy (**c**), and lifetime (**d**) of the Venus multimers. Each sample consisted of 5 replicates of 3 different cell homogenates, and black bar is the mean value. The relationship between normalized brightness (**e**) or fluorescence lifetime corrected normalized brightness (**f**) is plotted as a function of the number of Venus fluorophores in each construct. Each point is mean ± SD (n = 15). Red line represents a linear fit through the data, and dashed blue lines are the 95% confidence interval.

Our experiments indicate that auto-FPFA can be used to differentiate between mVenus monomers and dimers based on brightness, *τ*_D_, and anisotropy (compare Figs 3 and 4). Furthermore, anisotropy measurements can also be used to differentiate between dimers with subtle differences in separation. To further demonstrate the utility of using auto-FPFA to differentiate constructs with different numbers of fluorophores, we used auto-FPFA to compare V1, V2 (V5V), V4, and V6, Venus constructs with 1, 2, 4, and 6 Venus fluorophores respectively (Fig. 5). As the number of Venus molecules in a construct increases the molecular brightness of those constructs increases (Fig. 5a). Similarly, the diffusion time (*τ*_D_) also increased with the number of Venus molecules in a construct (Fig. 5b), presumably because the increase in the mass and changes in the hydrodynamic volume with increasing numbers of concatenated Venus molecules slows down lateral diffusion^16^. Steady-state anisotropy decreased with the number of Venus molecules in a construct (Fig. 5c), likely because homo-FRET mediated energy migration between multiple Venus fluorophores increases the extend of depolarization^16^. A small (179 ± 19 ps, n = 15) but statistically significant decrease (P < 0.0001 by ANOVA) in the Venus lifetime was observed when comparing the mVenus monomer (3.148 ± 0.003 ns, V1) and the Venus hexamer (2.969 ± 0.019 ns, V6) (Fig. 5d). Smaller reductions in the mVenus lifetime were observed for V2 (65 ± 4 ps) or V4 (138 ± 7 ps). Because the instrument response function of the hybrid-detectors used are ~140 ps, it is prudent to be skeptical of lifetime changes of the same magnitude or smaller. Nonetheless, because of the high level of reproducibility observed in auto-FPFA lifetime measurements, as well as the added statistical power derived from having 15 replicates, the systematic attenuation in the Venus lifetime appears to be a valid observation. Presumably, these small changes in lifetime are caused by changes in the local refractive index^37–40^.

Brightness analysis is a useful tool for measuring the stoichiometry of protein complexes^16,17,19,41–43^. Typically, the molecular brightness of an assembly of subunit, each tagged with a fluorophore, is divided by the molecular brightness of the fluorophore alone to determine how many fluorophore-tagged subunits are in the assembly. This is called the ‘*normalized brightness*’, ***ρ***. In this analysis, the normalized brightness value should be equal to the average number of fluorophore-tagged subunits in an assembly.

1 $$\rho =\frac{{\eta }_{c}}{{\eta }_{m}}$$

where ***η***_c_ is the molecular brightness of the complex in cpms, ***η***_m_ is the molecular brightness of the monomer, and ***ρ*** is the normalized brightness. The value of ***ρ*** should be an integer for complexes with a fixed number of subunits from a homogeneous population. In contrast, a non-integer value for ***ρ*** is indicative of heterogeneity in the population with regard to their subunit stoichiometry.

Equation 1 assumes that the fluorescence lifetime is the same in the complex and the monomer. If these lifetimes are different, Equation 2 can be used to compensate for those changes.

2 $$\rho =\frac{{\eta }_{c}\cdot \,\frac{\langle {\tau }_{m}\rangle }{\langle {\tau }_{c}\rangle }}{{\eta }_{m}}\,$$

where <*τ*_m_> is the amplitude-weighted average fluorescence-lifetime of the monomer, in this case V1, and <*τ*_c_> is the amplitude weighted average lifetime of the complex. We compared the validity of using these equations for measuring stoichiometry using auto-FPFA. The normalized brightness of the samples depicted in Fig. 5a were plotted as a function of the number of Venus fluorophores per molecule using either Equation 1 (Fig. 5e) or 2 (Fig. 5f). While both equations revealed a linear relationship between normalized brightness and the number of fluorophores in a construct, the slope of a linear fit using Equation 1 was 0.95 (95% confidence interval 0.9252–0.9791) while the slope measured using Equation 2 was 1.02 (95% confidence interval (0.9712–1.074). While subtle, it is clear that the normalized brightness of the V4 and V6 samples are under-estimated when the change in lifetimes were not accounted for (compare Fig. 5e,f). Note that Equation 2 reduces to Equation 1 when <*τ*_m_> = <*τ*_c_>.

### Testing a Hetero-FRET sensor

**Figure 6:**
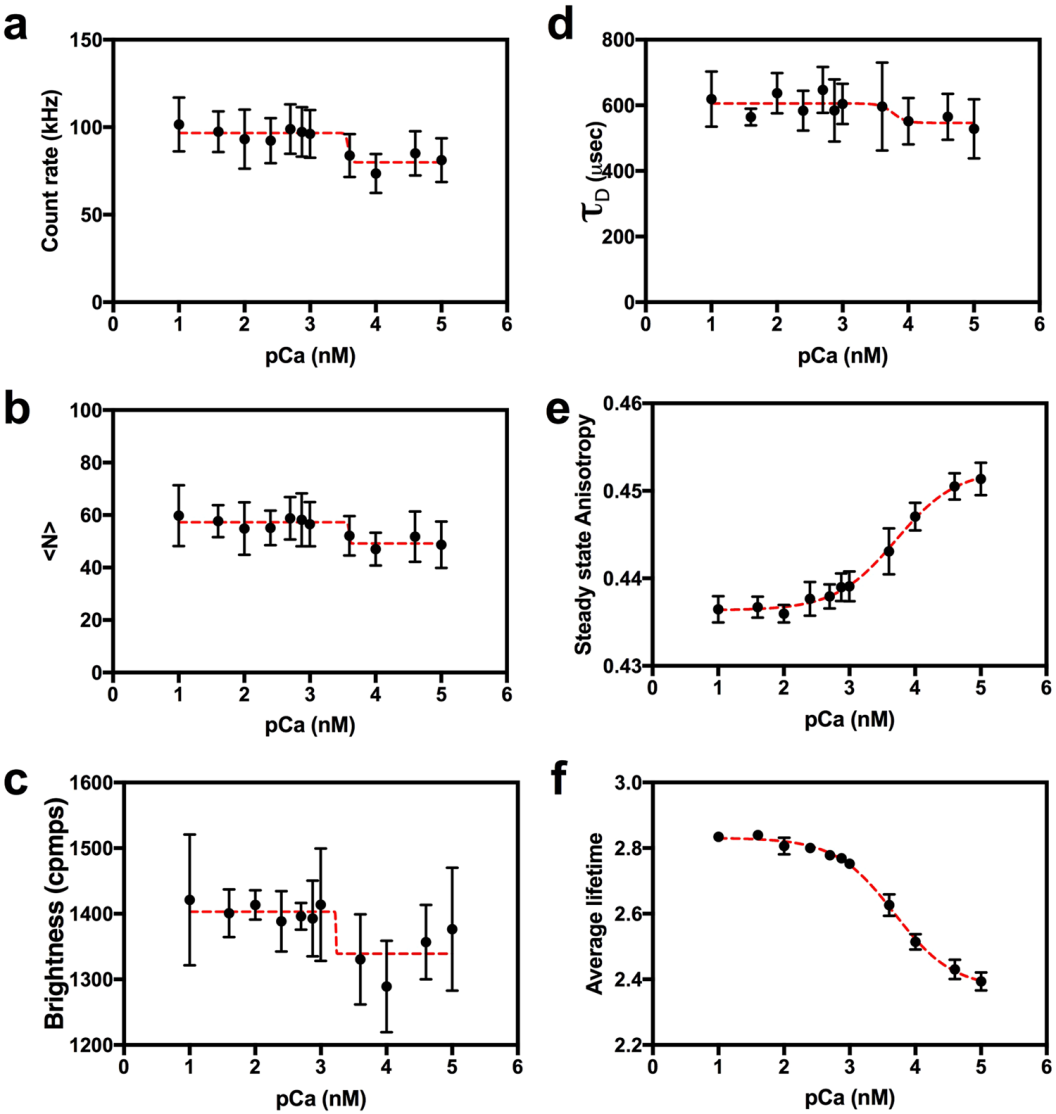
Auto-FPFA characterization of Venus-Twitch4-mCherry, a hetero-FRET based calcium biosensor. Auto-FPFA was used to measure count rate (**a**), <N> (**b**), brightness (**c**), diffusion time (*τ*_D_, **d**), steady state anisotropy (**e**), and average lifetime (**f**) of two different homogenates prepared from cells expressing Venus-Twitch4-mCherry. Homogenates were diluted into buffers with defined calcium concentrations where pCa indicates the log of the free calcium concentration in nano-moles. Each point is the mean ± SD (N = 4, two from each homogenate preparations). Dashed lines are a four parameter, variable slope dose-response fit.

Twitch-4 is a low-affinity genetically-encoded biosensor that monitors calcium concentration with an apparent K_d_ of 2.8 µM, and a Hill coefficient of 1.04^44^. Twitch-4 is a FRET based biosensor thought to have one active and one inactive EF-hand calcium binding site. The calcium binding domain is flanked by an ECFP FRET donor and a cpCit174 FRET acceptor. Because our microscope was optimized for use with yellow fluorescent proteins, we re-engineered Twitch-4 by replacing the ECFP with mVenus, and by replacing cpCit174 with mCherry. We call this new variant *V-Twitch-4-mCh*. We next characterized V-Twitch-4-mCh by performing a calcium dose response study using auto-FPFA (Fig. 6). We observed calcium dependent changes in every parameter measured by auto-FPFA. We measured a 16.7 ± 5.1% drop in the Venus emission count rate from 96.0 ± 2.4 to 80.0 ± 4.5 kHz (mean ± SD) occurring in buffers having between 1–4 µM calcium (Fig. 6a). This is consistent with a 16.7 ± 5.1% change in FRET efficiency upon binding calcium. The average number of fluorescent molecules in the observation volume, <N> as measured by fluctuation analysis dropped by ~14.1% from 57.3 to 49.2 (Fig. 6b). This also occurred between 1–4 µM calcium. Surprisingly we also observed a small decrease in the molecular brightness occurring between 1–4 µM calcium (Fig. 6c). Because errors were large for both the <N> and molecular brightness measurements relative to the signal it is not clear if these changes are significant. The *τ*_D_ for V-Twitch-4-mCh also dropped at high calcium concentrations, from 605.6 ± 14.4 to 546.5 ± 27.0 µs, but interestingly this transition occurred between 4–10 µM calcium (Fig. 6d). *τ*_D_ is sensitive to changes in the hydrodynamic volume of the molecule, thus this change suggests Twitch-4 contracts when calcium is bound. While *τ*_D_ is sensitive to lateral diffusion, steady-state anisotropy reports on changes in rotational diffusion. We observed an increase in the V-Twitch-4-mCh steady-state anisotropy from 0.4363 ± 0.0005 to 0.4524 ± 0.0012 with an apparent K_d_ of 5.1 µM (pCa = 3.7 ± 0.1) and a Hill slope of 0.94 ± 0.18 (Fig. 6e). Thus, despite Twitch-4 becoming more compact upon binding calcium, the Venus fluorophore attached to Twitch-4 apparently rotates slower when calcium is bound. The average fluorescent lifetime of V-Twitch-4-mCh decreased from 2.831 ± 0.007 to 2.373 ± 0.014 ns upon binding calcium with an apparent K_d_ of 4.7 µM (pCa = 3.68 ± 0.05) and a Hill slope of 0.97 ± 0.08 (Fig. 6f). This represents a 16.2 ± 5.4% drop in lifetime. Using the average lifetime of Venus monomers (3.154 ± 0.005 ns) for FRET efficiency measurements, this transition corresponds to a change in FRET efficiency of 14.6 ± 0.5%. Thus, calcium dependent changes in the V-Twitch-4-mCh Venus count rate and its lifetime mirror each other and both reflect energy transfer from mVenus to mCherry. These changes occur with a decrease in both lateral diffusion time and Venus rotational diffusion time. Thus, on a molecular scale, V-Twitch-4-mCh compacts when it binds calcium and this conformational change constrains the ability of the attached Venus to rotate.

### Testing a Homo-FRET sensor

**Figure 7:**
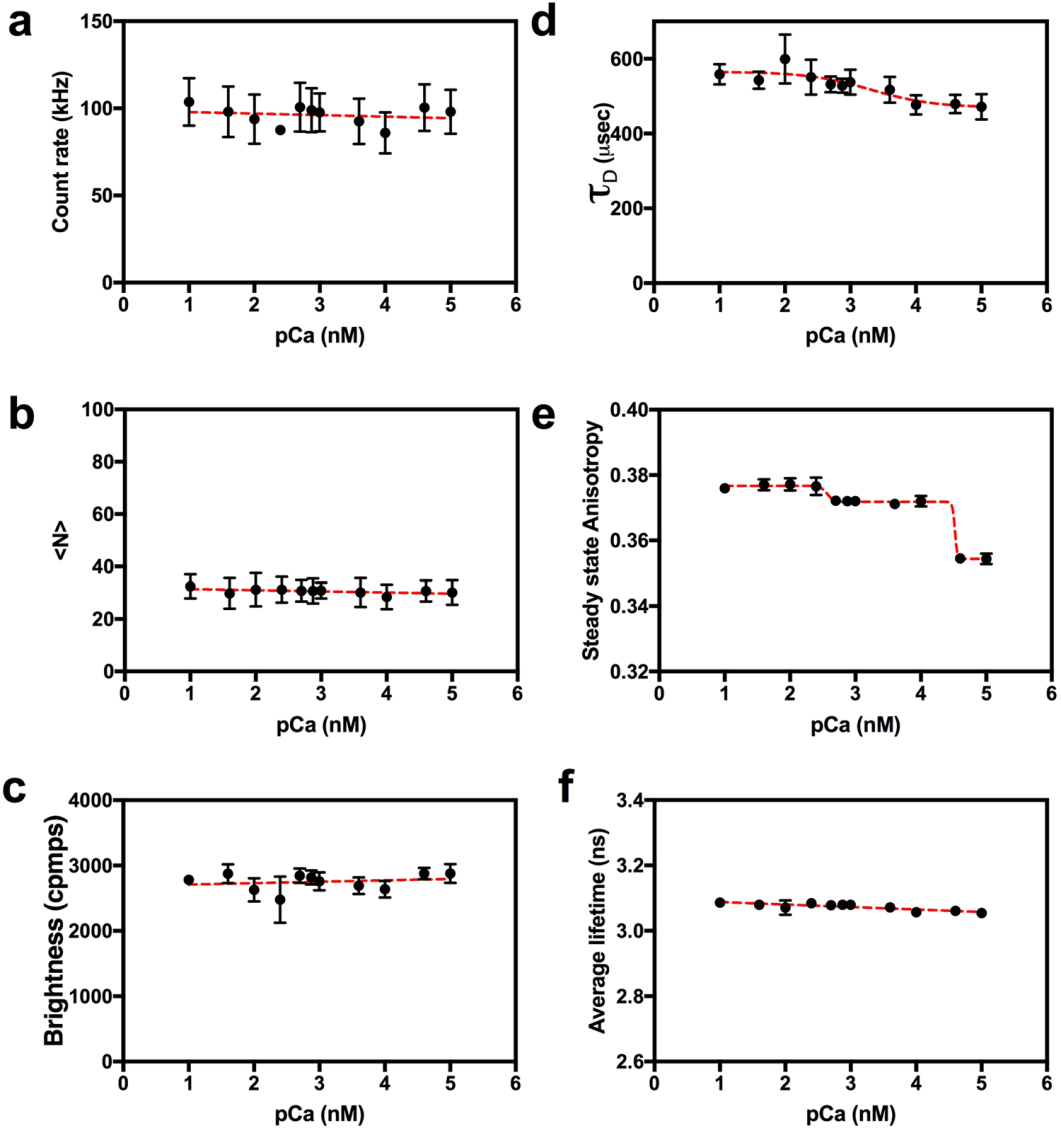
Auto-FPFA characterization of Venus-Twitch4-Venus, a homo-FRET based calcium biosensor. Auto-FPFA was used to measure count rate (**a**), <N> (**b**), brightness (**c**), diffusion time (*τ*_D_, **d**), steady state anisotropy (**e**), and average lifetime (**f**) of two different homogenates prepared from cells expressing Venus-Twitch4-Venus. Homogenates were diluted into buffers with defined calcium concentrations. Each point is the mean ± SD (N = 4, two from each homogenate preparations). Dashed lines are: a linear fit (panels a, b, c and f), a four parameter, variable slope dose-response fit (panel d) or a biphasic dose-response fit (panel e).

We next replace mCherry in V-Twitch-4-mCh with mVenus to create a homo-FRET based calcium sensor (V-Twitch-4-V) Increased homo-FRET should cause a decrease in anisotropy. Thus, based on the lifetime change observed in V-Twitch-4-mCh we expect that V-Twitch-4-V should have a large drop in its anisotropy around 4.7 µM calcium. Auto-FPFA analysis of V-Twitch-4-V revealed that unlike V-Twitch-4-mCh, it’s count rate, <N>, brightness, and lifetime does not change with calcium (Fig. 7a–c,f). The *τ*_D_ for V-Twitch-4-V dropped at high calcium concentrations, from 565.2 ± 14.1 to 469.6 ± 19.9 µs (Fig. 7d) with a K_d_ of 2.1 µM (pCa = 3.3 ± 0.4) and a Hill coefficient of 0.9 ± 0.6. Surprisingly, while V-Twitch-4-V did have a calcium dependent drop in its anisotropy, this drop was biphasic occurring between 0.25–0.5 µM calcium and between 10–40 µM calcium (Fig. 7e). The high affinity drop represented 22% of the total change in anisotropy, while the low affinity accounted for 78%. This suggests that Twitch-4 has two functional EF hand binding sites, one high affinity and one low. Complicating the interpretation of this anisotropy signal is the fact that anisotropy signals are functions of both changes in homo-FRET and changes in the molecular rotation of it’s two Venus molecules. Thus, the biphasic anisotropy change observed in Fig. 7e reflects cumulative effects from homo-FRET and molecular rotation, most likely in the opposite direction.

### Negative control

**Figure 8:**
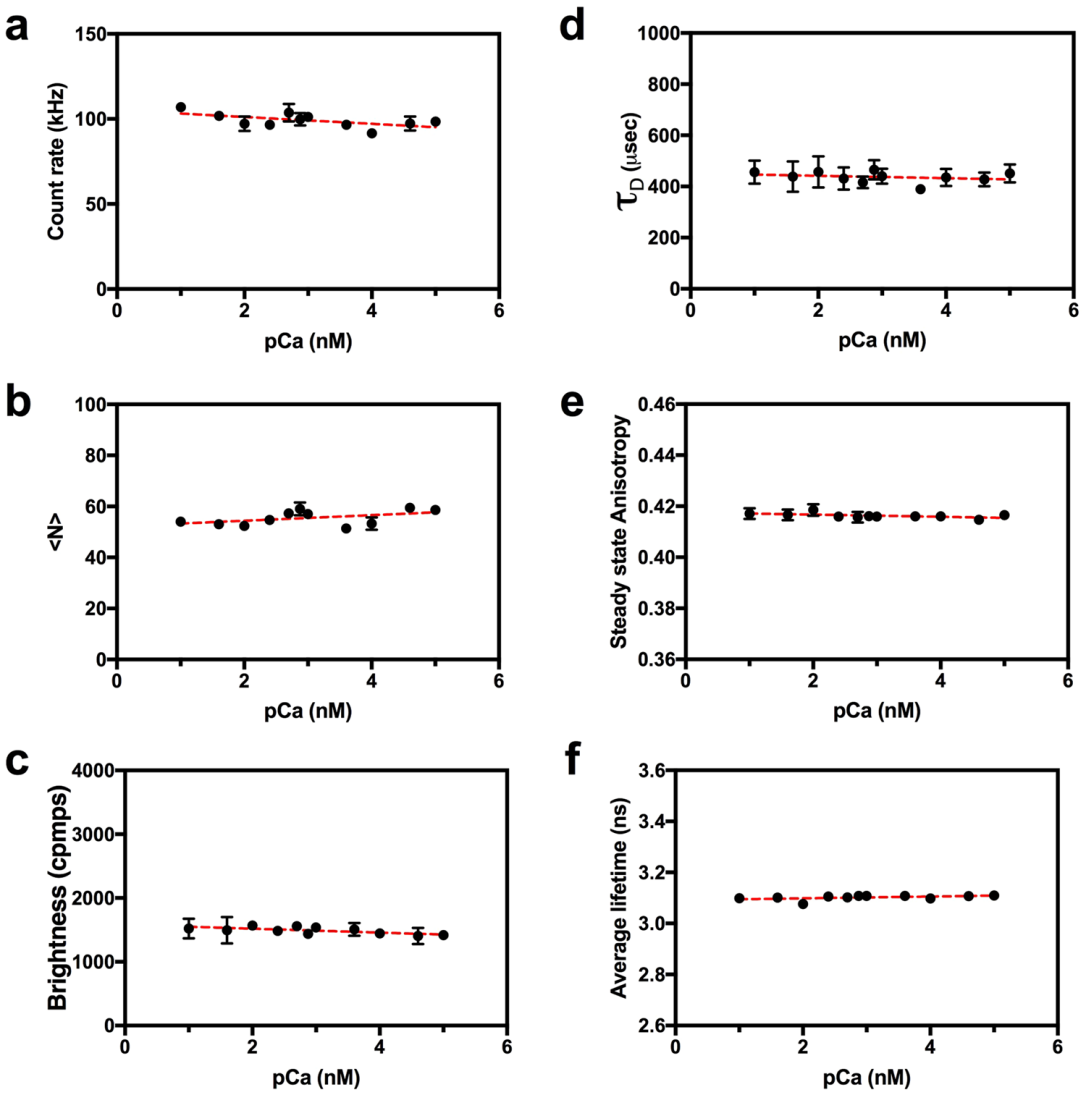
**A**uto-FPFA characterization of Venus monomers, a control for non-specific calcium changes in Venus photo-physics. Auto-FPFA was used to measure count rate (**a**), <N> (**b**), brightness (**c**), diffusion time (*τ*_D_, **d**), steady state anisotropy (**e**), and average lifetime (**f**) of two different homogenates prepared from cells expressing Venus. Homogenates were diluted into buffers with defined calcium concentrations. Each point is the mean ± SD (N = 4, two from each homogenate preparations). Dashed lines are a linear fit.

We ran a calcium dose-response for mVenus as a negative control (Fig. 8). As expected, there was no obvious change in *τ*_D_ (437 ± 21 µs, mean ± SD), steady state anisotropy (0.42 ± 0.00), and lifetime (3.10 ± 0.01 ns) as free calcium was increased, and these values were comparable to our pervious measurements of mVenus in calcium free buffer (see Fig. 3). While the mVenus molecular brightness, count rate and <N> values also did not change as a function of calcium (Fig. 8a–c), the average brightness value (1489 ± 56 cpms) was higher than in Fig. 3a (843 ± 70 cpms), because higher laser powers were used in the calcium dose-response experiment depicted in Figs 6–8.

### Auto-FPFA calcium dose-response of GCaMP6s

GCaMP6s is a fluorescent calcium sensor, derived from GFP, whose emission intensity increases with elevated calcium (K_d_ = 144 nM)^45^. At low calcium, one of the staves of the ß-barrel structure protecting the GCaMP6s fluorophore is destabilized reducing fluorescence, while at high calcium the ß-barrel structure is stabilized resulting in bright fluorescence. It was specifically designed to emit fluorescence, albeit dimly, even at low calcium concentrations, to aid in identifying cells expressing GCaMP6s at low resting calcium levels. The photo-physical basis of this dim calcium independent emission is poorly understood. To better understand the molecular basis for the GCaMP6s biosensor we characterized its fluorescence signal using auto-FPFA.

**Figure 9:**
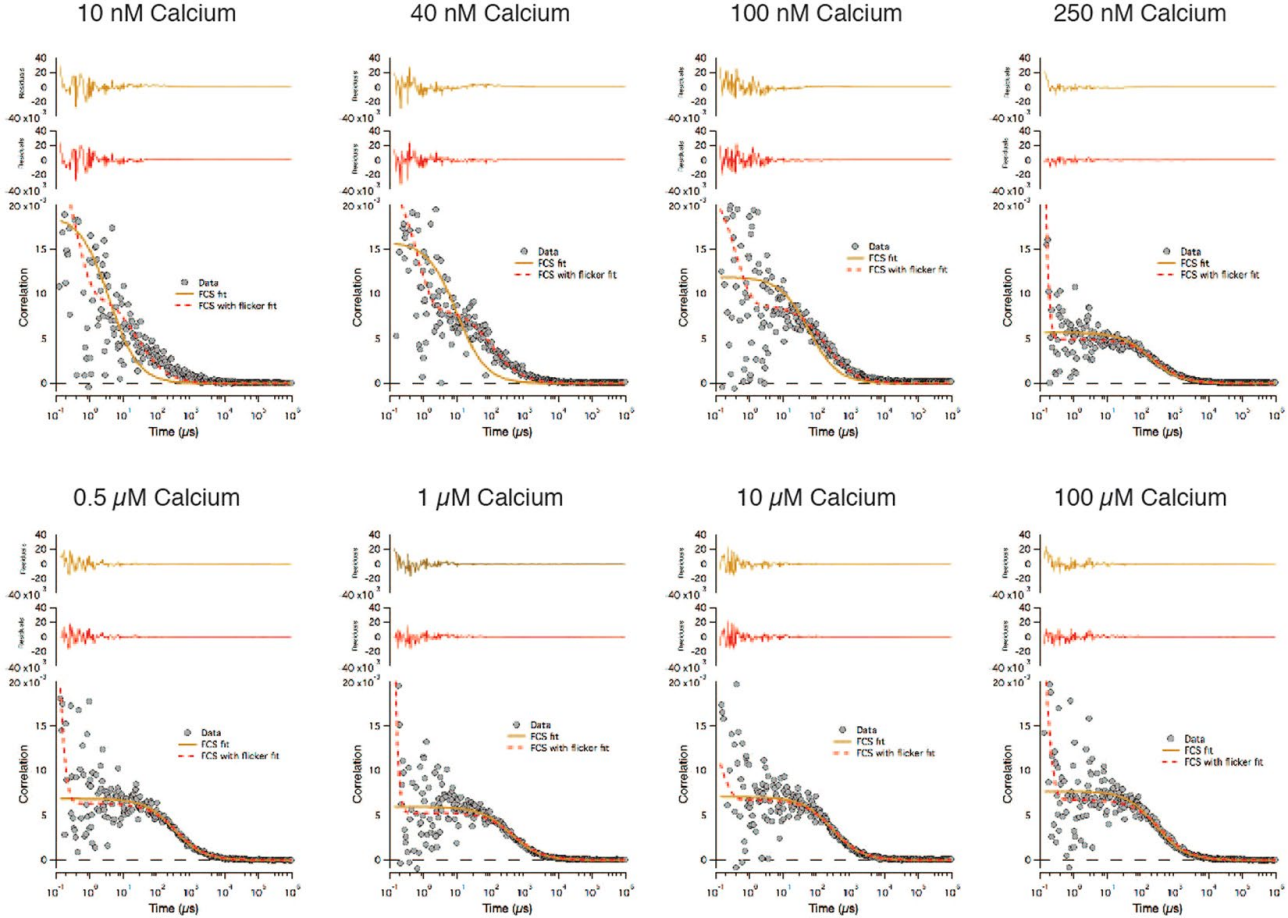
Auto-FPFA cross-correlation analysis of GCaMP6s as a function of free-calcium concentration. The FCS component of auto-FPFA was used to measure cross-correlation data of homogenates prepared from cells expressing GCaMP6s. Representative cross-correlation curves are plotted at different calcium concentrations. Homogenates used at 10, 40, 100 and 250 nM calcium was six times as concentrated as those used at 0.5, 1, 10, and 100 µM calcium. Each auto-FPFA cross-correlation curve was fit to either a simple single diffusion component FCS model (FCS fit, solid yellow traces) or a more complex FCS model that allows for the diffusion of a single fluorophore that can ‘flicker’, i.e. transition between a dark and bright state (FCS with flicker fit, dashed red traces). Residuals for each fit are plotted. Note that at low calcium concentrations the simple FCS fit model fails to fit the data properly.

Unlike the other calcium biosensors analyzed, we found that GCaMP6s auto-FPFA required using two different concentrations of sample to accommodate its stringent requirements for the concentrations of fluorescent molecules in the observation volume. For calcium concentration at or below 250 nM we used approximately six times the concentration of GCaMP6s as was used for calcium concentration above 250 nM. While cross-correlation curves for V-Twitch-4-mCh and V-Twitch-4-V were well fit to a single component FCS diffusion model (FCS fit) at all calcium concentrations (data not shown), cross-correlation curves of GCaMP6s at calcium concentrations below 250 nM were poorly fit by this model, but were well fit using a more complex model that includes fluorophore *flickering* (FCS with flicker fit) (Fig. 9). Flickering is a photo-physical phenomenon where a fluorophore reversibly transitions between a dark state and its fluorescent state^46,47^. This model adds two additional fitting parameters to the FCS model, the fraction of molecules in the dark state, and *τ*_F_, the average amount of time a molecule remains in the bright state. GCaMP6s cross-correlation curves were well fit using the simple single-component FCS diffusion model from calcium concentrations at 250 nM or higher.

**Figure 10:**
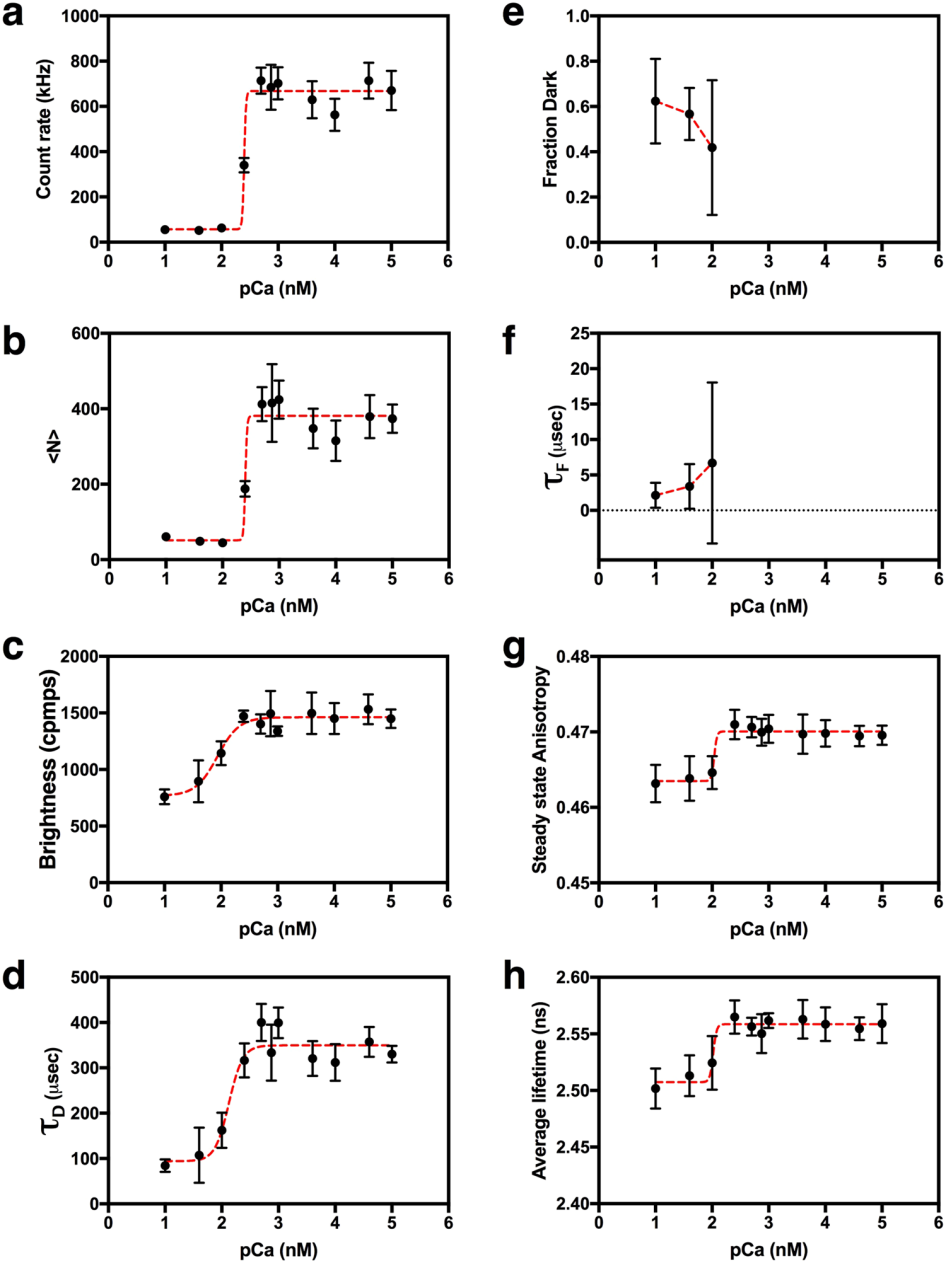
Auto-FPFA characterization of GCaMP6s, an intensity based calcium biosensor. Auto-FPFA was used to measure count rate (**a**), <N> (**b**), brightness (**c**), *τ*_D_ (**d**), fraction dark state (**e**), *τ*_F_ (**f**), steady state anisotropy (**g**), and average lifetime (**e**) of two different homogenates prepared from cells expressing GCaMP6s. Homogenates were diluted into buffers with defined calcium concentrations. The homogenate concentration was six times as concentrated for samples at or below 250 nM calcium as those used at higher calcium concentrations. Each point is the mean ± SD (N = 4, two from each homogenate preparations). Dashed lines are a four parameter, variable slope dose-response fit in all panels except for panels e and f where the dashed lines simply connect the three data points.

As expected, auto-FPFA revealed that GCaMP6s count rate increased sharply between 100–250 nM free calcium (Fig. 10a). This increase was mimicked by the calcium dependence of <N> (Fig. 10b). GCaMP6s molecular brightness doubled with increased calcium (Fig. 10c), suggesting that calcium triggered GCaMP6s dimerization might occur. This however seems unlikely as homo-FRET between GCaMP molecules was not observed (Fig. 10g). In fact, anisotropy increased with calcium, suggesting that calcium binding causes an increase in the GCaMP6s rotational correlation time. Interestingly, the increase in molecular brightness occurred prior to any change in count rate indicating that quenching of the GCaMP6s chromophore at low concentrations of calcium might be occurring. GCaMP6s chromophore quenching was corroborated by fluorescent lifetime measurements (Fig. 10h). One explanation for quenching of the GCaMP6s chromophore is that as the ß-barrel structure of GCaMP6s is destabilized at low calcium concentrations, quenchers can gain access to its fluorophore. Indeed, the presence of flicker at low calcium concentrations (Fig. 9), the observation that the fraction of molecules in a dark state decreases as the calcium concentration increases (Fig. 10e), while the average amount of time that GCaMP6s can emit photons (*τ*_F_) increases (Fig. 10f), all support this explanation.

One puzzling finding revealed by auto-FPFA is that the value of *τ*_D_ from FCS analysis also increased as a function of calcium from 94 ± 18 to 350 ± 9 µs with an apparent K_D_ of 134 nM calcium (Fig. 10d). When using a simple single component diffusion model for FCS analysis, *τ*_D_ is interpreted as the *diffusion time*, the average time that a fluorescent molecule spends in the excitation volume. Diffusion time is usually a function of the molecules hydrodynamic volume, its mass, and the solvents viscosity. GCaMP6s is a construct composed of a circularly permutated GFP molecule with a calcium sensing adduct. Thus, we expect GCaMP6s to have a diffusion time similar to, if not larger, than a Venus monomer (Fig. 3b, 383 ± 61 µs), as is seen at calcium concentrations at and above 250 nM. How then, is it possible for GCaMP6s to have a *τ*_D_ value below 100 µs at low calcium? One possibility is that GCaMP6s has a very compact structure at low calcium. Because *τ*_D_ is relatively insensitive to changes in mass or hydrodynamic volume, a 3.7-fold decrease in diffusion time seems unlikely. We note that the GCaMP6s reduction in *τ*_D_ was only observed in samples displaying high levels of flicker, which required the use of the more complicated flicker model for fitting. While the flicker model fit GCaMP6s data adequately, the fit is based on the assumption that the molecule flickers between two states, completely dark, or completely bright. Our observation of intermediate values for molecular brightness (Fig. 10c) and lifetime (Fig. 10h) indicate that more complex fitting models might be warranted to better understand the molecular basis for GCaMP6s calcium sensing.

## Discussion

**Figure 11:**
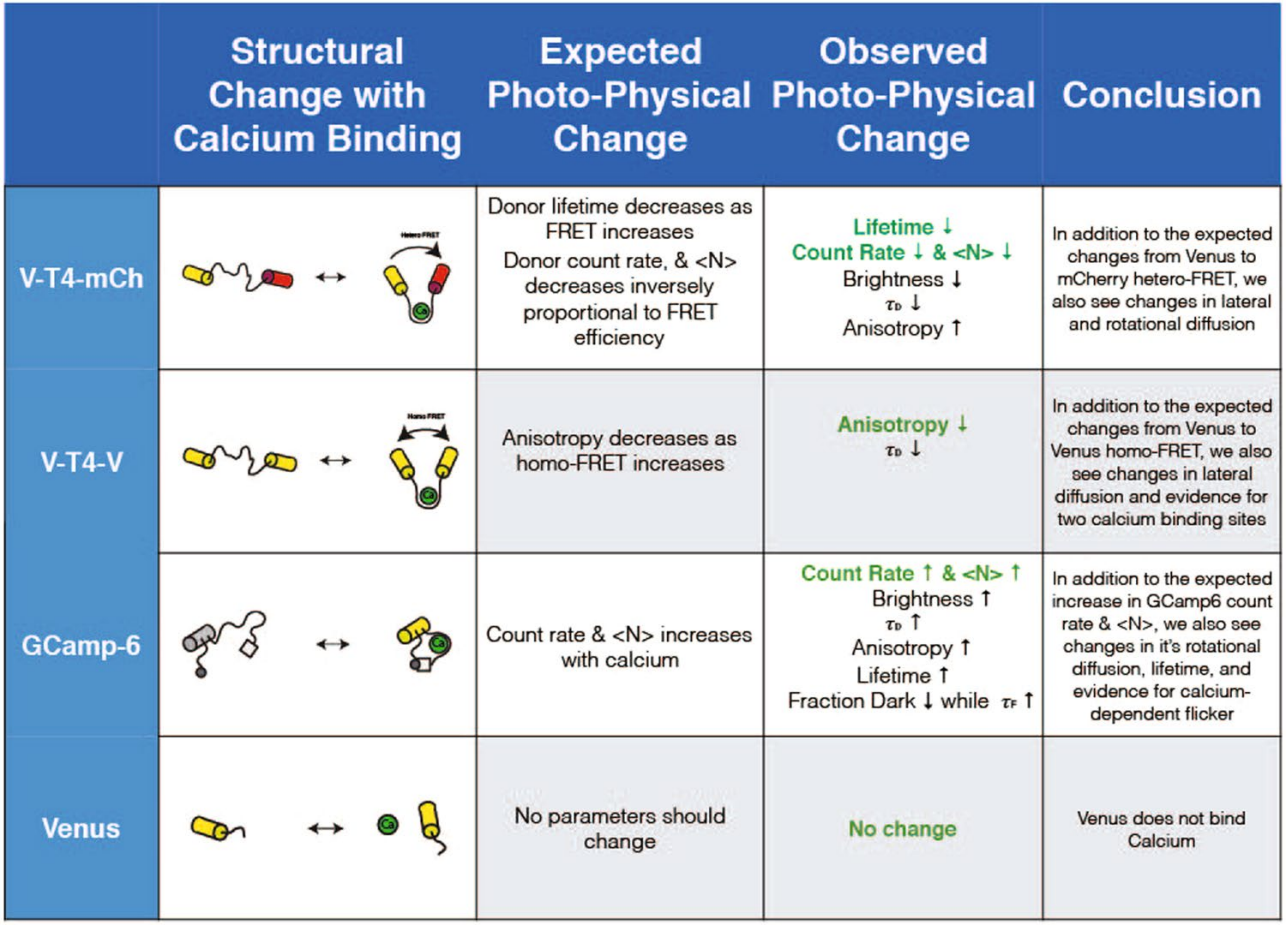
Data synopsis of auto-FPFA of three calcium sensors and negative control. Cartoon at left depicts the structural changes thought to occur upon calcium binding. Table depicts the expected photo-physical changes and the observed changes in the constructs investigated.

We have automated FPFA microscopy to allow the autonomous measurement of intensity, fluorescence lifetime, time-resolved anisotropy, molecular brightness, lateral diffusion time, and <N> from up to 96 different samples. We validated this approach using Venus monomers (Fig. 3), Venus dimers with different separation (Fig. 4), and Venus concatemers (Fig. 5). Next, we demonstrated the utility of auto-FPFA for characterizing genetically encoded biosensors by performing calcium dose-response analysis on three different types of calcium biosensors (Fig. 11), one based on monitoring changes in fluorescence lifetime (Fig. 6), one based on monitoring changes in fluorescence anisotropy (Fig. 7), and one based on monitoring changes in intensity (Fig. 10). Venus monomers served as a negative control for structural changes caused by calcium binding to the fluorophore (Fig. 8). We note that while GCaMP has been extensively used in neuroscience to detect neuronal activity^48–50^, V-Twitch-4-mCh and V-Twitch-4-V are new spectral variants of the Twitch-4 biosensor^44^ that have never been characterized before. The auto-FPFA data presented in Figs 6 and 7 were derived from only two replicate auto-FPFA runs. Figure 11 summarizing the photo-physical changes expected and observed for these biosensors. It is also worth noting that for each biosensor studied, photo-physical changes, other than those expected from the biosensor design, were observed. This observation is not unexpected, but we feel it is underappreciated. One of the challenges of designing biosensors is first finding a photo-physical trait that changes with ligand binding, and then optimizing the biosensor to maximize the change in the observable. Often, simply confirming that a construct can bind the ligand of interest may be a challenge. For example, it is well known that the absence of FRET does not indicate that a donor and acceptor are not interacting^51–54^. Thus, a biosensor characterized by only monitoring FRET might fail to identify constructs that can bind the ligand. Because auto-FPFA monitors intensity, lifetime, anisotropy, brightness, diffusion time, and concentration, it is unlikely that a fluorescent construct could interact with a ligand and not alter at least one of these observables.

In addition to its utility for characterizing biosensors, auto-FPFA may also be useful for structure-function studies of large protein assemblies^17–19^, and mutagenesis studies^17^. For these types of studies, it is important to appreciate FPFA’s limitations. Because it requires samples in the 10–100 nM range low affinity complexes might dissociate at these concentrations. Finally, this stringent concentration requirement, in addition to the presence of large intracellular structures capable of disrupting FCS measurements, makes live-cell auto-FPFA measurements impractical.

## Materials and Methods

### Cell culture, transfection and homogenate preparation

TSA 201 cells (ATCC) were cultured as a monolayer in a T-75 Flask (CytoOne) in a humidified incubator (Thermo Scientific) containing 9% CO_2_ in air at 37 °C in DMEM (1X) + GlutaMAX^TM^-1 media containing D-Glucose, sodium pyruvate and 10% fetal bovine serum (all from Gibco). A day prior to FPFA measurement, cells were suspended using TrypLE Express (Gibco by Life Technologies) and washed with DPBS (without calcium and magnesium, Gibco by Life Technologies). For *in vitro* measurements, plasmid DNA encoding Venus-tagged constructs (typically 1 µg/250,000 cells) were transfected into the cells using electroporation (Digital Bio/BTX MicroPorator). Transfected cells were plated on 60 mm culture dishes (Corning) and incubated overnight. On the following day, cells were harvested and lysed using passive lysis buffer (Promega). Homogenates were centrifuged at 100,000 × g for 30 minutes to remove membranes and particulate matter. Supernatants were diluted for FPFA to yield a photon count rate between ~20,000 cps (>25x the dark count rate) and <100,000 cps (to avoid TCSPC pile-up artifacts^55^). Clarified homogenates (200 μl) were then loaded into 96-well glass bottom plates (Greiner Bio-one) and measured by Auto-FPFA on the same day. For calcium dose-response measurements, samples of interest were equally diluted into 11 calcium buffers with concentration ranging from 10 nM to 100 μM (Calbuf-2, World Precision Instruments).

### Molecular Biology

Venus monomer (mVenus-C1), Venus dimers (V5V also called V2, V17V, V32V) and Venus multimers Venus tetramer (V4), Venus hexamer (V6) were previously described and are available at Addgene^56,57^.

Addgene Identifiers:

Venus (mVenus-C1): Plasmid #27794

V5V: Plasmid #29423

V17V: Plasmid #29424

V32V: Plasmid #29561

V4 (VVVV): Plasmid #29425

V6 (VVVVVV): Plasmid #27813

Venus-Twitch 4-mCherry was generated in two steps. First the mCherry open reading frame was amplified from pmCherry-NLS (a gift from Martin Offterdinger, Addgene plasmid # 39319) using Q5 polymerase (NEB) and cloned into the EcoRI and BamHI sites of Venus-C1. The Twitch-4 calcium binding domain from Twitch-4 pcDNA3 (a gift from Oliver Griesbeck, Addgene plasmid # 49533) was amplified with flanking BspEI and EcoRI sites using Q5 polymerase and cloned between the Venus-mCherry open reading frames.

Venus-Twitch 4-Venus was generated in three steps. First a codon degenerate version of mVenus was designed to avoid issued with future PCR based manipulations caused by open reading frame sequence redundancy. The sequence for this construct (VenusHP) was generated (GenScript) by codon optimization (humanized), synthesized and provided cloned into the XbaI and ApaI sites of pcDNA3.1. The synthetic construct included an in frame SacII site between the XbaI site and a strong Kozak sequence (GCCACCATG). The open reading frame from mVenus-C1 was removed with NheI and HindIII and cloned into the same sites in the VenusHP vector. The Twitch-4 calcium binding domain from Twitch-4 pcDNA3 was amplified with flanking BspEI and SacII sites using Q5 polymerase and cloned between the Venus-VenusHP open reading frames.

pGP-CMV-GCaMP6s was a gift from Douglas Kim (Addgene plasmid # 40753).

### Time resolved fluorescence anisotropy and lifetime analysis

The fluorescence decay of parallel and perpendicular channels were used to calculate the time-resolved anisotropy using the following equation^25,57^:

3 $$r(t)=\frac{{I}_{\parallel }(t)-g.{I}_{\perp }(t)}{{I}_{\parallel }(t)+2.g.{I}_{\perp }(t)}$$

where I_II_(t) and I_⊥_(t) are fluorescence intensity of parallel and perpendicular channels (dark noise subtracted) respectively, and g is the instrument correction factor which for our microscope had a value of 1.07 as determined by calibration using fluorescein tail fitting. Similarly, steady state anisotropy was also estimated from parallel and perpendicular fluorescence decays:

4 $$R=\frac{\int {I}_{\parallel }(t)-g\cdot \int {I}_{\perp }(t)}{\int {I}_{\parallel }(t)-2\cdot g\cdot \int {I}_{\perp }(t)}$$

The time-dependent fluorescence intensity decay was calculated using the following relationship^55^:

5 $$I(t)={I}_{\parallel }(t)+2.g.{I}_{\perp }(t)$$

For most samples, *lifetimes* were calculated by fitting the time-dependent fluorescence intensity decay to a single exponential decay model. For samples where hetero-FRET between Venus and mCherry fluorophores occurs, their more complex multi-exponential time-dependent fluorescence intensity decay was fit to a double exponential decay model, and the amplitude-weighted *average lifetime* was calculated based on the following equation:

6 $$\langle \tau \rangle =\frac{{a}_{1}.{\tau }_{1}+{a}_{2}.{\tau }_{2}}{{a}_{1}+{a}_{2}}$$

where *a*_1_ & *a*_2_ *are* the amplitudes of the decay component and *τ*_1_ & *τ*_2_ are their decay constants.

### Fluorescence fluctuation analysis of polarized channels

A cross-correlation curve is generated from I_||_(t) and I_⊥_ (t) fluctuations, is fitted to a single component three-dimensional Gaussian diffusion model^58^, *G(τ*), to estimate the values <N>, the average number of fluorescent molecules in the observation volume, and τ_D_, the correlation time, typically interpreted as the average time that a molecule spends in the detection volume:

7 $$G(\tau )=\frac{\gamma }{\langle N\rangle }\frac{1}{1+(\tau /{\tau }_{D})}\frac{1}{\sqrt{1+{(\omega /z)}^{2}(\tau /{\tau }_{D})}}$$

where ω and z, are the radial and axial beam waists respectively, and the constant γ has a value of 0.35 for a two-photon three-dimensional Gaussian PSF^58^.

The molecular brightness η is the average number of photon emitted per second per molecule (cpms):

8 $$\eta =\frac{\langle k\rangle }{\langle N\rangle }$$

where <*k>* is the average photon count rate recorded during data acquisition.

The normalized brightness, ρ, of a Venus-tagged protein complex was determined by dividing the molecular brightness (η_c_) of a complex composed of Venus-tagged subunits, by the molecular brightness of a Venus monomer (η_m_):

9 $$\rho =\frac{{\eta }_{c}}{{\eta }_{m}}$$

Note that η_c_ and η_m_ should be measured using similar conditions (primarily using the same laser excitation power, filters, and optics), and that η_m_ can be measured by running a Venus monomer control.

If the Venus lifetimes is altered in a construct, the following equation was used to compensate for those changes.

10 $$\rho =\frac{{\eta }_{c}\cdot \frac{\langle {\tau }_{m}\rangle }{\langle {\tau }_{c}\rangle }}{{\eta }_{m}}\,$$

where <*τ*_m_> is the amplitude-weighted average fluorescence-lifetime of the Venus monomer, and <*τ*_c_> is the amplitude weighted average lifetime of the complex.

The relationship between correlation time, τ_D_, and the diffusion coefficient, D, with two-photon excitation is given by^28^:

11 $${\tau }_{D}=\frac{{\omega }^{2}}{8D}$$

### Flickering analysis

Cross-correlation curves of GCaMp6s samples displaying flicker at low calcium concentrations were fit to a single-component three-dimensional Gaussian diffusion model with flicker^59^:

12 $$G(\tau )=\frac{\gamma }{\langle N\rangle }\cdot \frac{1}{1+(\tau /{\tau }_{D})}\cdot \frac{1}{\sqrt{1+{(\omega /z)}^{2}(\tau /{\tau }_{D})}}\cdot \frac{1-T+T.{e}^{t/{\tau }_{T}}}{1-T}$$

where T is the fraction of fluorescent molecules that transitions into a dark state, and *τ*_*T*_ is the *flicker time* whose reciprocal is referred as the rate at which the dark state population is populated.

### Auto-FPFA Calibration

The instrument correction factor *g* for calculating time-resolved anisotropy (Equation 3) was measured using tail-fitting^25^ of fluorescein samples and found to be 1.07. At high pH, fluorescein has a constant quantum yield and its diffusion coefficient D is 436 μm^2^/s at room temperature^60^. Thus, using fluorescein as a diffusion standard, Equation 7 can be used to estimate the value of ω (at a specific excitation power) by measuring fluorescein’s correlation time (at the same power). For example, using D = 436 μm^2^/s, and a measured correlation times of 70.1 ± 4.8 µs (n = 15), the value of ω was 494 ± 17 nm with 9.6 mW excitation power (at 950 nm, after the objective). The ratio ω/z (Equations 7 and 12) was measured by global fitting (to Equation 7) of cross-correlation curves obtained from known dilutions of fluorescein. In this calibration, it is assumed that with constant excitation power for all fluorescein dilutions, only the value <N> will change with dilution. At 9.6 mW excitation power, the ω/z ratio was 0.049 ± 0.02, and taken together with our estimate for ω predicts a z value of 10.1 ± 4.1 µm. The validity of this calibration procedure was confirmed by measuring the diffusion coefficient of Venus monomers under identical conditions. Using ω = 494 ± 17 nm and the measured correlation time for Venus monomers with 9.6 mW excitation power of 377 ± 40 µs (n = 15), the diffusion coefficient for Venus monomers in solution was 81 ± 10 µm^2^/s (n = 15), in excellent agreement with the diffusion coefficient measured for GFP (78.4 ± 6.4 µm^2^/s)^61^. The two-photon observation volume (V) at any specific excitation power can be calculated using the following equation^58^:

13 $$V=\frac{{\pi }^{3/2}{\omega }^{2}z}{8}$$

Accordingly, the observation volume with 9.6 mW excitation power was 1.7 ± 0.7 fl. Note that this volume, and the value of <N> from a FPFA measurement can be used to calculate the concentration of Venus or of Venus-tagged protein complexes, a key factor for determining if non-specific inter-molecular-FRET (due to molecular crowding) can occur.

### Curve fitting and statistics

IGOR Pro software (WaveMetrics Inc., Ver. 7.06) was used to develop an analysis tool for standard and global fitting of time-resolved fluorescence intensity, time-resolved anisotropy and cross-correlation curves of multiple samples. GraphPad Prism 7 was used to calculate means and standard deviations (SD), and linear fits for brightness controls. Values are presented throughout the text as mean ± SD, deviations of ±0.00 indicate a value of less than 0.005. GraphPad Prism was also used to calculate ANOVA test.

### Data availability

The datasets generated during and/or analysed during the current study are available from the corresponding author on reasonable request.

## References

[1] Lakowicz, J. R. *Principles of Fluorescence Spectroscopy*. Third edn, (Springer, 2006).

[2] Valeur, B. *Molecular Fluorescence* (Wiley-VCH, 2002).

[3] Jameson DM, Croney JC, Moens PD (2003). Fluorescence: basic concepts, practical aspects, and some anecdotes. Methods Enzymol.

[4] Weidtkamp-Peters S (2009). Multiparameter fluorescence image spectroscopy to study molecular interactions. Photochem Photobiol Sci.

[5] Chalfie M, Tu Y, Euskirchen G, Ward WW, Prasher DC (1994). Green fluorescent protein as a marker for gene expression. Science.

[6] Heim R, Prasher DC, Tsien RY (1994). Wavelength mutations and posttranslational autoxidation of green fluorescent protein. Proc Natl Acad Sci USA.

[7] Patterson GH, Knobel SM, Sharif WD, Kain SR (1997). & Piston, D. W. Use of the green fluorescent protein and its mutants in quantitative fluorescence microscopy. Biophys J.

[8] Zhang J, Campbell RE, Ting AY, Tsien RY (2002). Creating new fluorescent probes for cell biology. Nat Rev Mol Cell Biol.

[9] Shaner NC, Steinbach PA, Tsien RY (2005). A guide to choosing fluorescent proteins. Nat Methods.

[10] Miyawaki A (1997). Fluorescent indicators for Ca2+ based on green fluorescent proteins and calmodulin. Nature.

[11] Suhling K (2004). Time-resolved fluorescence anisotropy imaging applied to live cells. Opt Lett.

[12] Guzman C, Oetken-Lindholm C, Abankwa D (2016). Automated High-Throughput Fluorescence Lifetime Imaging Microscopy to Detect Protein-Protein Interactions. J Lab Autom.

[13] Margineanu A (2016). Screening for protein-protein interactions using Forster resonance energy transfer (FRET) and fluorescence lifetime imaging microscopy (FLIM). Scientific reports.

[14] Talbot CB (2008). High speed unsupervised fluorescence lifetime imaging confocal multiwell plate reader for high content analysis. J Biophotonics.

[15] Schaufele F (2014). Maximizing the quantitative accuracy and reproducibility of Forster resonance energy transfer measurement for screening by high throughput widefield microscopy. Methods.

[16] Nguyen TA, Sarkar P, Veetil JV, Koushik SV, Vogel SS (2012). Fluorescence polarization and fluctuation analysis monitors subunit proximity, stoichiometry, and protein complex hydrodynamics. PLoS One.

[17] Sarkar P (2017). Deciphering CaMKII Multimerization Using Fluorescence Correlation Spectroscopy and Homo-FRET Analysis. Biophysical Journal.

[18] Nguyen TA (2015). Covert Changes in CaMKII Holoenzyme Structure Identified for Activation and Subsequent Interactions. Biophys J.

[19] Kraft LJ, Nguyen TA, Vogel SS, Kenworthy AK (2014). Size, stoichiometry, and organization of soluble LC3-associated complexes. Autophagy.

[20] Elson EL, Madge D (1974). Fluorescence Correlation Spectroscopy. I. Conceptual Basis and Theory. Biopolymers.

[21] Magde D, Elson EL, Webb WW (1974). Fluorescence correlation spectroscopy. II. An experimental realization. Biopolymers.

[22] Elson EL, Webb WW (1975). Concentration correlation spectroscopy: a new biophysical probe based on occupation number fluctuations. Annu Rev Biophys Bioeng.

[23] Gautier I (2001). Homo-FRET microscopy in living cells to measure monomer-dimer transition of GFP-tagged proteins. Biophys J.

[24] Tramier M, Coppey-Moisan M (2008). Fluorescence anisotropy imaging microscopy for homo-FRET in living cells. Methods Cell Biol.

[25] Vogel, S. S., Thaler, C., Blank, P. S. & Koushik, S. V. In *FLIM* Microscopy *in Biology and Medicine* (eds Periasamy, A. & Clegg, R. M.) Ch. 10, 245–290 (Taylor & Francis, 2010).

[26] Vogel, S. S., Nguyen, T. A., Blank, P. S. & Wieb Van Der Meer, B. In *Springer Series in Chemical Physics Advanced Time-Correlated Single Photon Counting Applications* Ch. 12, 624 (Springer, 2015).

[27] Thompson, N. L. In *Topics in Fluorescence Spectroscopy* Vol. 1 (ed Lakowicz, J. R.) Ch. 6, 337–378 (Plenum Press, 1991).

[28] Berland KM, So PT, Gratton E (1995). Two-photon fluorescence correlation spectroscopy: method and application to the intracellular environment. Biophys J.

[29] Schwille, P., Korlach, J. & Webb, W. W. Fluorescence correlation spectroscopy with single-molecule sensitivity on cell and model membranes. *Cytometr*y **3**6, 176–182, 10.1002/(SICI)1097-0320(19990701)36:3176::AID-CYTO53.0.CO;2-F (1999). 10.1002/(sici)1097-0320(19990701)36:3<176::aid-cyto5>3.0.co;2-f 10404965

[30] Chirico G, Olivini F, Beretta S (2000). Fluorescence excitation volume in two-photon microscopy by autocorrelation specroscopy and photon counting histograms. Applied Spectroscopy.

[31] Hess ST, Webb WW (2002). Focal volume optics and experimental artifacts in confocal fluorescence correlation spectroscopy. Biophys J.

[32] Berland K, Shen G (2003). Excitation saturation in two-photon fluorescence correlation spectroscopy. Appl Opt.

[33] Axelrod D (1979). Carbocyanine dye orientation in red cell membrane studied by microscopic fluorescence polarization. Biophys J.

[34] Axelrod D (1989). Fluorescence polarization microscopy. Methods Cell Biol.

[35] Zipfel WR, Williams RM, Webb WW (2003). Nonlinear magic: multiphoton microscopy in the biosciences. Nat Biotechnol.

[36] Nagai T (2002). A variant of yellow fluorescent protein with fast and efficient maturation for cell-biological applications. Nat Biotechnol.

[37] Suhling K (2002). Imaging the environment of green fluorescent protein. Biophys J.

[38] Toptygin D, Savtchenko RS, Meadow ND, Roseman S, Brand L (2002). Effect of the Solvent Refractive Index on the Excited-State Lifetime of a Single Tryptophan Residue in a Protein. J. Phys. Chem. B.

[39] Suhling K, French PM, Phillips D (2005). Time-resolved fluorescence microscopy. Photochem Photobiol Sci.

[40] Koushik SV, Vogel SS (2008). Energy migration alters the fluorescence lifetime of Cerulean: implications for fluorescence lifetime imaging Forster resonance energy transfer measurements. J Biomed Opt.

[41] Chen Y, Wei LN, Muller JD (2005). Unraveling protein-protein interactions in living cells with fluorescence fluctuation brightness analysis. Biophys J.

[42] Chen Y, Muller JD (2007). Determining the stoichiometry of protein heterocomplexes in living cells with fluorescence fluctuation spectroscopy. Proc Natl Acad Sci USA.

[43] Chen Y, Johnson J, Macdonald P, Wu B, Mueller JD (2010). Observing protein interactions and their stoichiometry in living cells by brightness analysis of fluorescence fluctuation experiments. Methods Enzymol.

[44] Thestrup T (2014). Optimized ratiometric calcium sensors for functional in vivo imaging of neurons and T lymphocytes. Nat Methods.

[45] Chen TW (2013). Ultrasensitive fluorescent proteins for imaging neuronal activity. Nature.

[46] Schwille P, Kummer S, Heikal AA, Moerner WE, Webb WW (2000). Fluorescence correlation spectroscopy reveals fast optical excitation-driven intramolecular dynamics of yellow fluorescent proteins. Proc Natl Acad Sci USA.

[47] Dittrich P, Malvezzi-Campeggi F, Jahnz M, Schwille P (2001). Accessing molecular dynamics in cells by fluorescence correlation spectroscopy. Biol Chem.

[48] Cui G (2013). Concurrent activation of striatal direct and indirect pathways during action initiation. Nature.

[49] Tian L (2009). Imaging neural activity in worms, flies and mice with improved GCaMP calcium indicators. Nat Methods.

[50] Akerboom J (2012). Optimization of a GCaMP calcium indicator for neural activity imaging. J Neurosci.

[51] Medintz, I. & Hildebrandt, N. *FRET – Förster Resonance Energy Transfer*. (Wiley-VCH Verlag GmbH & Co. KGaA, 2013).

[52] Clegg, R. M. in *FRET an*d FLI*M* Techn*iques* Vol. 33 *Laboratory Techniques in Biochemistry and Molecular Biology* (ed Gadella, T. W. J.) Ch. 1, 1–57 (Elsevier, 2009).

[53] Periasamy, A. & Clegg, R. M. FLIM Microscopy in Biology and Medicine. First edn, (Taylor & Francis, 2010).

[54] Periasamy, A., Mazumder, N., Sun, Y., Christopher, K. G. & Day, R. N. In *Springer Series in Chemical Physics Advanced Time-Correlated Single Photon Counting Applications* Ch. 7, 624 (Springer, 2015).

[55] Becker, W. *Advanced Time-Correlated Single Photon Counting Techniques* (Springer, 2005).

[56] Koushik SV, Chen H, Thaler C, Puhl HL, Vogel SS (2006). Cerulean, Venus, and VenusY67C FRET reference standards. Biophys J.

[57] Thaler C, Koushik SV, Puhl HL, Blank PS, Vogel SS (2009). Structural rearrangement of CaMKIIalpha catalytic domains encodes activation. Proc Natl Acad Sci USA.

[58] Muller JD, Chen Y, Gratton E (2003). Fluorescence correlation spectroscopy. Methods Enzymol.

[59] Widengren J, Mets Ü, Rigler R (1995). Fluorescence correlation spectroscopy of triplet states in solution: a theoretical and experimental study. J Phys Chem A.

[60] Petrasek Z, Schwille P (2008). Precise measurement of diffusion coefficients using scanning fluorescence correlation spectroscopy. Biophys J.

[61] Chen Y, Muller JD, Ruan Q, Gratton E (2002). Molecular brightness characterization of EGFP in vivo by fluorescence fluctuation spectroscopy. Biophys J.

